# Structure and electrical properties of sputtered TiO_2_/ZrO_2 _bilayer composite dielectrics upon annealing in nitrogen

**DOI:** 10.1186/1556-276X-7-31

**Published:** 2012-01-05

**Authors:** Ming Dong, Hao Wang, Cong Ye, Liangping Shen, Yi Wang, Jieqiong Zhang, Yun Ye

**Affiliations:** 1 State Key Laboratory of Electrical Insulation and Power Equipment, School of Electrical Engineering, Xi'an Jiaotong University, Xi'an, Shanxi, 710049, China; 2 Faculty of Physics and Electronic Technology, Hubei University, Wuhan, 430062, China

## Abstract

The high-*k *dielectric TiO_2_/ZrO_2 _bilayer composite film was prepared on a Si substrate by radio frequency magnetron sputtering and post annealing in N_2 _at various temperatures in the range of 573 K to 973 K. Transmission electron microscopy observation revealed that the bilayer film fully mixed together and had good interfacial property at 773 K. Metal-oxide-semiconductor capacitors with high-*k *gate dielectric TiO_2_/ZrO_2_/p-Si were fabricated using Pt as the top gate electrode and as the bottom side electrode. The largest property permittivity of 46.1 and a very low leakage current density of 3.35 × 10^-5 ^A/cm^2 ^were achieved for the sample of TiO_2_/ZrO_2_/Si after annealing at 773 K.

## Introduction

High dielectric constant [high-*k*] materials have been researched for a few years in material science and have been applied firstly in Intel's 45 nm MOSFET in 2007. Nowadays, for the demand of the next generation devices for sub-22 nm technology nodes, expect that high-*k *materials such as HfO_2_, ZrO_2_, Ta_2_O_5_, and rare earth oxides are extensively researched, and binary oxides of high-*k *materials become more attractive and are expected to be utilized in the future ultra large scale integrated circuit [1-8]. Among them, ZrO_2 _has a relatively high permittivity, large band gap, and good thermal and chemical stabilities. TiO_2 _is a high-*k *material with a very high permittivity of about 80 [9]. In order to improve the permittivity of ZrO_2_, the feasible way is to fabricate ZrO_2_-TiO_2 _composite films. Meanwhile, as a composite thin film, the addition of TiO_2 _can improve the crystallization temperature [10,11]. As ZrO_2_-TiO_2 _binary oxides, a nanolaminate structure which can tailor the electrical properties of dielectric stacks has many applications such as MIM diodes, storage capacitors, non-volatile memories, and transparent thin film transistors; thus, the nanolaminated ZrO_2_-TiO_2 _high dielectric constant thin film is worth studying

Concerning high-*k *stacks on silicon, the interface has an important role to influence the device. Normally, it is often thought that TiO_2 _is easier to react with the Si substrate which may deteriorate the property of the device, and thus, TiO_2_/ZrO_2_/Si stacks may have better electrical characterization [12-14]. In the present work, metal-oxide-semiconductor [MOS] capacitors with high-*k *gate dielectric TiO_2_/ZrO_2_/p-Si were fabricated using Pt as the top gate electrode and as the bottom side electrode. The structure and electrical property of the TiO_2_/ZrO_2_/Si stack are studied.

## Experimental details

ZrO_2 _and TiO_2 _thin films were grown onto p-type (100) Si (P~10^15 ^cm^-3^) to fabricate TiO_2_/ZrO_2_/Si stacks by radio frequency magnetron sputtering at room temperature. Pure ZrO_2 _(99.999%) and TiO_2 _(99.999%) ceramic targets (50 mm in diameter) were used as the sputtering targets. The sputtering power of ZrO_2 _and TiO_2 _are 60 W and 30 W, respectively. Pure argon (99.999%) with 30 cm^3^/min flow rate controlled by a mass flow controller was used as sputtering gas, and the base pressure of the vacuum chamber is about 3 × 10^-5 ^Pa. Sputtering was carried out at a pressure of 0.3 Pa. As for the deposited TiO_2_/ZrO_2_/Si stacks, post annealing of 573 K, 773 K, and 973 K in N_2 _for 30 min was performed.

The structural characteristics of the films were investigated by X-ray diffraction [XRD] (Bruker D8, Bruker, Billerica, MA, USA) and transmission electron microscopy [TEM] (FEI Tecnai G20, FEI Co., Hillsboro, OR, USA). Film thickness was determined by an *ex situ *phase-modulated spectroscopic ellipsometry [SE] (Model Jobin Yvon, HORIBA Jobin Yvon Inc., Edison, NJ, USA) over the spectral range of 1.5 to 6.5 eV at an angle of incidence of 70°. For the purpose of exploring electrical properties, a Pt/TiO_2_/ZrO_2_/p-Si MOS capacitor was fabricated by sputtering a Pt top electrode with an area of 1.96 × 10^-7 ^m^2 ^through a shadow mask. The back side of the wafer was HF-cleaned, and the Pt thin film was deposited. The MOS capacitors were electrically characterized using a Radiant Precision Premier (Radiant Technologies Inc., Albuquerque, NM, USA) tester system to obtain current-voltage [*I*-*V*] curves. Capacitance-voltage [*C*-*V*] measurements were performed by a precision LCR meter (Agilent 4294A; Agilent Technologies Inc., Santa Clara, CA, USA).

## Results and discussion

The chemical composition of the TiO_2_/ZrO_2_/Si film can be measured by XRF, and all samples have nearly the same atomic Ti content of 21%, which indicates that the annealing process did not change the composition. Concerning the Ti content in the TiO_2_-ZrO_2 _binary system, the optimal content of about 21% has been verified in our previous work [10].

Spectroscopic ellipsometry was employed to measure the film thickness. The Tauc-Lorentz model which is especially suitable for an amorphous material was adopted to characterize the dielectric function of the TiO_2_/ZrO_2 _bilayer composite film [15-17]. In order to get the best fitting of SE data, different models were built due to the structure change of the TiO_2_/ZrO_2 _bilayer composite film. For the as-deposited thin film, a double layer optical model was built on Si (100) substrate, i.e., ZrO_2 _layer (L_1_) and TiO_2 _layer (L_2_), while for the annealed one, only one layer of the ZrO_2_-TiO_2 _composite thin film was built. Lastly, we can obtain the thickness of the as-deposited thin film with a ZrO_2 _layer (L_1_) of 27.639 ± 0.521 nm and TiO_2 _layer (L_2_) of 10.077 ± 0.627 nm. For the sample annealed at 773 K, the total thickness is 28.149 ± 1.102 nm. This result indicates that annealing makes the film denser and decreases the thickness.

**Figure 1:**
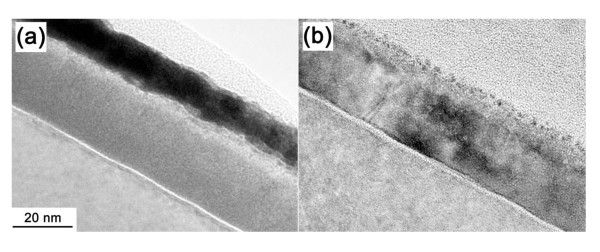
**Cross-sectional TEM images of TiO_2_/ZrO_2_/Si thin films**. (**a**) As-deposited and (**b**) annealed at 773 K.

The detailed structure of the TiO_2_/ZrO_2_/Si film was studied by TEM. We take the as-deposited and 773 K annealed samples representatively for analysis. Figure 1a presents the micrograph of the as-deposited sample. It can be clearly seen that the structure includes the two layer stacks of TiO_2 _and ZrO_2 _films and that the interface layer is observed between the ZrO_2 _film and the Si substrate. The physical thickness of ZrO_2 _and TiO_2 _thin films was measured to be 26 and 13 nm, respectively, which is consistent with the SE measurement. Figure 1b shows the cross-sectional image of the 773 K annealed TiO_2_/ZrO_2 _thin film. Obviously, after 773 K annealing, the two-layer structure became one layer for the mixture of TiO_2 _and ZrO_2_. It is reported that the multilayer film often fully mixed at 773 K [18]. The thickness from TEM can be calculated to be 30 nm and agrees well with the fitting result from SE.

**Figure 2:**
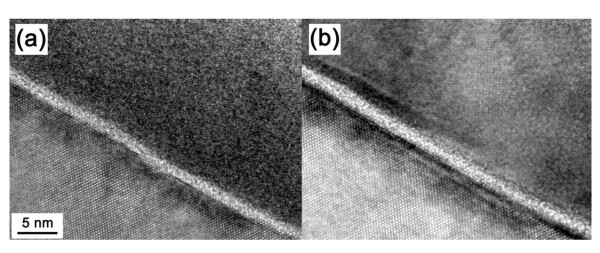
**High-resolution cross-sectional TEM images of the interface between the composite thin films and Si**. (**a**) As-deposited and (**b**) annealed at 773 K.

Figure 2 presents the high-resolution TEM images of the interface property of the TiO_2_/ZrO_2_/Si films. It is believed that the interface layers play an important role on the electrical properties, including the dielectric constant and the leakage currents. From Figure 2, it can be seen that there is no obvious difference for the as-deposited and 773 K annealed samples. Both interface have a thickness of about 1.1 nm. We consider it to be SiO_2 _appearing at the ZrO_2_/Si interface. The relatively thin interface layer of 1.1 nm can be regarded as a good interfacial property for the TiO_2_/ZrO_2_/Si film. TEM also shows that both films are either amorphous or amorphous-like structures with a little nanocrystalline part in the 773 K annealed samples. This result can be confirmed by XRD, where the as-deposited thin film and the annealed ones are amorphous (XRD not shown here).

**Figure 3:**
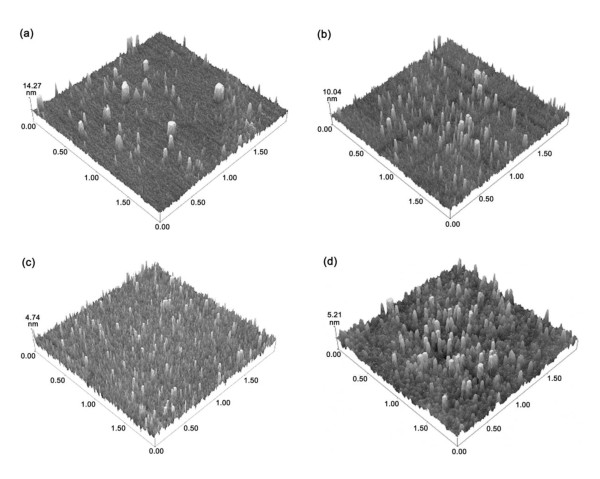
**AFM images of TiO_2_/ZrO_2_/Si thin films**. (**a**) As-deposited, (**b**) annealed at 573 K, (**c**) annealed at 773 K, and (**d**) annealed at 973 K.

Figure 3 shows the atomic force microscopy [AFM] images of the TiO_2_/ZrO_2 _thin films. One can clearly see that the surface morphology of the films depends on the annealing temperature. The RMS roughness of the as-deposited film and annealed ones was measured over a 2 × 2 μm^2 ^scanning range, and the values are 1.430, 1.529, 0.625 and 0.826 nm, respectively. One can see that the surface roughness of the thin film decreases at higher annealing temperature. At 773 K annealing temperature, the film has the smallest surface roughness, which may be attributed to the full incorporation of the TiO_2 _and ZrO_2 _film, as shown in TEM.

**Figure 4:**
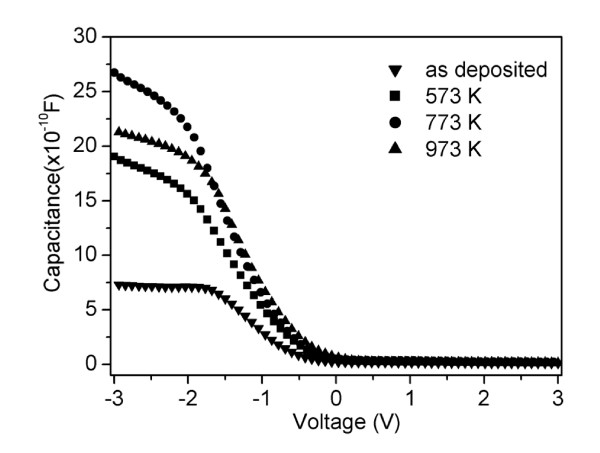
**High-frequency (1 MHz) capacitance-voltage curves for TiO_2_/ZrO_2_/Si thin films**. The inverted triangle represents the as-deposited sample; square, the sample annealed at 573 K; circle, the sample annealed at 773 K; and triangle, the sample annealed at 973 K.

*C*-*V *characteristics of the MOS capacitor consisting of Pt/TiO_2_/ZrO_2_/p-Si was measured at high frequency (1 MHz). Figure 4 shows the *C*-*V *curves for the ZrO_2_/TiO_2 _thin films. It can be seen that at 773 K annealed temperature, the saturated capacitance is the highest. According to the saturated capacitance, we can get the effective dielectric constant of the thin films. The dielectric constants of annealed composite thin films are much higher than those of the pure ZrO_2 _(about 20) [19], which indicate that TiO_2 _has been incorporated in the ZrO_2 _film and improved the overall *k *value. Meanwhile, the dielectric constants of the annealed samples are higher than the as-deposited one, which is only 16.6 and can be attributed to the series capacity of the two-layer structure [20]. At 773 K, the dielectric constant of the composite thin film is the highest and reaches the maximum of 46.1, while at 973 K, the dielectric constant decreases to be 36.9. It can be concluded that the dielectric constants are affected by the annealing temperature. Normally, for a composite thin film, the dielectric constant is mainly dependent on the component of the film and the microstructure including the crystalline property, interface, surface roughness, and various vacancies and defects in the film, etc. [21-24]. At 773 K, based on the above analyses, the multilayer film fully mixed, has good interfacial property, has the smallest surface roughness, and has an amorphous structure, which results in the highest dielectric constant. The relatively small *k *of 33.0 at 573 K may result from the multilayer film that was only partly mixed although the film is amorphous. At 973 K, the decrease of dielectric constant is possibly due to interfacial reaction at high annealing temperature. We also obtain the flat band voltage [*V*_fb_] from the high frequency *C*-*V *curves. *V*_fb _primarily depends on deficiencies in theTiO_2_/ZrO_2 _film and the interface traps at the interface. The smallest *V*_fb _is -0.53 V for the 773 K annealed thin film, and for the as-deposited and 573 K and 973 K annealed samples, the values of *V*_fb _are *-*1.01, *-*0.71, and *-*0.62, respectively. It can be inferred from the *V*_fb _that annealing can reduce the deficiencies or traps in the composite TiO_2_/ZrO_2 _thin film and that the annealing temperature of 773 K is the optimal temperature.

**Figure 5:**
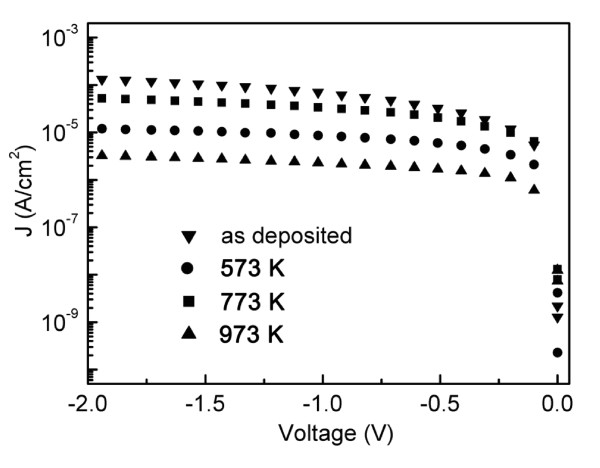
**Current-voltage curves for TiO_2_/ZrO_2_/Si thin films**. The inverted triangle represents the as-deposited sample; circle, the sample annealed at 573 K; square, the sample annealed at 773 K; and triangle, the sample annealed at 973 K.

Figure 5 shows the density-voltage [*J*-*V*] characteristics of all the samples with gate electron injection (negative *V*_g_). As shown in Figure 5, all the annealed samples have lower leakage current density than the as-deposited one for the reason that annealing makes the film denser and reduces defects in the film. For the 773 K annealed thin film, the leakage current density is about 3.35 × 10^-5 ^A/cm^2 ^at the applied voltage of -1 V, which is slightly higher than that of other high-*k *oxide materials. This may be caused by the interface layer as shown in Figure 2 and the defects in the film.

## Conclusion

The high-*k *dielectric TiO_2_/ZrO_2 _bilayer composite film was prepared on a Si substrate by radio frequency magnetron sputtering and post annealing in N_2 _at various temperatures in the range of 573 K to 973 K. The bilayer film fully mixed together to become a composite single layer and has good interfacial property after annealing at 773 K. The largest property permittivity of 46.1 and a low leakage current density of 3.35 × 10^-5^A/cm^2 ^were achieved for the sample of Pt/TiO_2_/ZrO_2_/Si/Pt after annealing at 773 K.

## Competing interests

The authors declare that they have no competing interests.

## Authors' contributions

MD carried out the electrical properties of TiO_2_/ZrO_2 _bilayer composite dielectrics and drafted the manuscript. HW conceived the study and participated in its design and coordination. CY participated in the revision of the manuscript. LPS and YW participated in the preparation of the TiO_2_/ZrO_2 _bilayer thin film. JQZ and YY contributed to the structure characterization of the TiO_2_/ZrO_2 _bilayer thin film. All authors read and approved the final manuscript.
